# A gold nanoparticles coated unclad single mode fiber-optic sensor based on localized surface plasmon resonance

**DOI:** 10.1038/s41598-023-32852-6

**Published:** 2023-04-07

**Authors:** Makram A. Fakhri, Evan T. Salim, Sara M. Tariq, Raed Khalid Ibrahim, Forat H. Alsultany, Ali. A. Alwahib, Sarmad Fawzi Hamza Alhasan, Subash C. B. Gopinath, Zaid T. Salim, U. Hashim

**Affiliations:** 1 Laser and Optoelectronic Engineering Department, University of Technology-Iraq, Baghdad, Iraq; 2 Applied science department, University of Technology-Iraq, Baghdad, Iraq; 3 AlFarahidi University, Baghdad, Iraq; 4 Department of Medical Physics, Al-Mustaqbal University College, Hillah, Iraq; 5 Department of Communication Engineering, University of Technology-Iraq, Baghdad, Iraq; 6 Institute of Nano Electronic Engineering, University Malaysia Perlis, 01000 Kangar, Perlis Malaysia; 7 Faculty of Chemical Engineering Technology, Universiti Malaysia Perlis, 02600 Arau, Perlis Malaysia; 8 Centre of Excellence for Nanobiotechnology and Nanomedicine (CoExNano), Faculty of Applied Sciences, AIMST University, 08100 Semeling, Kedah Malaysia

**Keywords:** Nanoscience and technology, Optics and photonics

## Abstract

In the last few decays, the fiber-optic was employed in the field of sensing because of its benefits in contrast to other types of sensors such as small size, easy to fabricate, high response, and flexibility. In this study, unclad single mode fiber-optic sensor is proposed to operate at 650 nm wavelength. COMSOL Multiphysics 5.1 finite element method (FEM) is used to design the sensor and tested it theoretically. The middle portion of the fiber cladding is removed and replaced by gold nanoparticles (Au NPs) of 50 nm thickness. Analytic layer of 3 μm thickness was immersed in different liquids in range of refractive index (RI) from 1.000281 to 1.39. These liquids are NaCl Deionized (DI) water solution, sucrose-Deionized (DI) water solution, and glycerol solution Deionized (DI) water. It was found that the highest obtained sensitivity and resolution are for glycerol-DI water solution with value of 3157.98 (nm/RIU) and 3.16 × 10^–5^ (RIU), respectively. Furthermore, it is easy to fabricate and of low cost. In experiments, pulsed laser ablation (PLA) was used to prepare Au NPs. X-ray diffraction (XRD) shown that the peak of the intensity grew as the ablated energy increased as well as the structure crystallization. Transmission electron microscopy (TEM) revealed an average diameter of 30 nm at the three ablated energies, while X-ray spectroscopy (EDX) spectrum has indicated the presence of Au NPs in the prepared solution. The photoluminescence (PL) and ultraviolet–visible UV–Vis transmission were used to study the optical properties of the prepared Au NPs. An optical spectrum analyzer was used to obtain the sensor's output results. It has shown that best intensity was obtained for sucrose which confined with theoretical results.

## Introduction

In the fields of biology, biomedical science, biochemistry, chemistry, and physics, there are numerous developments in the field of sensing. These advancements provided solutions to a number of sensor-related issues. These advancements provided solutions to a number of sensor-related issues. The issues that sensors may face include sensor size, ability to detect small variations, cost, amount of sample to be sensed, and performance parameters such as sensitivity, resolution, and signal to noise ratio…etc.^1–3^.

New insights into the control of various properties of nanomaterials that can support surface plasmons for specific applications have emerged as a result of recent advances in nanotechnology. The optical phenomenon of localized surface plasmon resonance (LSPR) is caused by a light wave trapped within conductive nanoparticles (NPs) with a wavelength shorter than that of light^4^.

The use of noble metal nanoparticle (NP) LSPR properties in conjunction with optical fibers to create localized surface plasmon resonance (LSPR) optical fiber sensors has become a popular topic of study. The localized electromagnetic field around metal surfaces is extremely sensitive to refractive index changes in the environment. Changes in metal LSPR characteristics can be used to track environmental changes at media-metal interfaces^5–7^.

LSPR sensors can be made with both multimode and single-mode optical fibers. The inherent modal noise limits the sensing resolution of the multimode fiber SPR sensor. The LSPR sensor is based on single-mode fiber to highlights the resolution^8–10^.

Over the last decade, laser-assisted fabrication of functional nanostructures has gotten a lot of attention. The synthesis technology of pulsed laser ablation in liquid (PLAL) is gradually gaining popularity because it is a chemically simple and clean method with high product purity. Furthermore, the method can be used in normal temperatures and pressures, allowing for easy control of product structures and properties by adjusting experimental conditions such as laser parameters, solutions, external environment, and target material. Once the pulsed laser with high energy is focused on the target in liquid, nanomaterial is easily fabricated. As a result of PLAL technology, various nanostructures with novel morphologies and special properties have been synthesized for applications in optics, biology, and energy^11–13^.

Raham et al. demonstrated a plastic optical fiber sensor based on Surface Plasmon Resonance (SPR) for estimating the concentration and refractive index of sugar in human blood serum. The refractive index increases as the resonance wavelength increases, as evidenced by the fact that at resonance wavelength (613 nm), the refractive index was (1.3628), which was increased by increasing the resonance wavelength until it reached its highest value of (1.387) at resonance wavelength (734 nm)^14^.

Kamrunnahar et al.^15^ investigated a simple circular lattice dual-core photonic crystal fiber (PCF) based plasmonic sensor by using FEM. The sensor operated in a range of wavelength extended from 450 to 1150 nm. In order to create SPR effect, gold is deposited on the outer surface of the proposed sensor. The sensor provided maximum wavelength sensitivity of 11500 nm/RIU and the amplitude sensitivity of 505.037RIU^−1^ with maximum Figure of Merit (FOM) of 275RIU^-1^ and resolution of 8.92 × 10^–6^ RIU within the RI range of 1.33 to 1.44.

Liu et al.^16^ proposed an enhanced plastic optical fiber (POF)-based surface plasmon resonance (SPR) sensor by employing a double-sided polished structure. When the measured liquid RI is 1.42, the double-sided POF-based SPR sensor has a sensitivity of 4284.8 nm/RIU. The proposed SPR sensor was simple to make and inexpensive.

Fakhri et al.^17^ used Au NPs as a plasmonic material to propose a modified (HC-800) Photonic Crystal Fiber (PCF) sensor by using FEM. The sensor provided maximum amplitude sensitivities of 769.5749, 975.5352, 869.8453 RIU^−1^.

However, the use of POF has disadvantages such as the high attenuation and dispersion, as well as incapability to outstand the high temperature. Furthermore, POF has inefficient sensitivity. In contrast, silica glass optical fiber, have higher sensitivity and optimized for small spacing and small targets. Thus, in this work, the cladding middle portion of single mode fiber SMF-28e is removed. Au NPs of thickness (50 nm) is deposited on the silica core (8.2 µm diameter) of fiber to enhance the sensitivity. Then, the unclad fiber is immersed in water, sucrose solution (30% concentration), glycerol (30% concentration), Sodium Chloride NaCl (30% concentration). The thickness of these liquids layer is (3 μm). This sensor provides a comparison between different liquids with different RIs. It has shown a sensitivity of 3157.98[nm/RIU] for glycerol-DI water solution with resolution of 3.16 × 10^–5^ [RIU] at RI of 1.39 and wavelength of 650 nm. All these steps are tested theoretically by using COMSOL Multiphysics 5.1 simulation program in order to see the possibility to achieve this sensor experimentally in real world.

In this work, The use of silica glass optical fiber shows more efficient sensitivity than plastic optical fiber POF that was used in previous research. Provides a comparison between different liquids with different refractive indices RIs. Provides experimental work and shows the possibility of achieving the sensor in the real world.

## Theory and simulation

We have proposed an optical fiber sensor based on LSPR phenomenon where single mode fiber SMF-28e is used where etching the middle portion of the fiber cladding. The core (8.2 µm diameter) and the cladding (125 µm diameter) are made of silica glass. In order to absorb a fraction of the core guided mode, the removed cladding is replaced by Au NPs (50nm thickness) which is coated on the core of the fiber to construct the fiber-optic sensor and enhance the sensitivity. Then, the fiber is immersed in different liquids (3 μm thickness) at 650 nm operating wavelength and RIs ranging from (1.000281-1.39). These liquids are water, sucrose solution (C_12_H_22_O_11_), glycerol solution (C_3_H_8_O_3_), and NaCl solution.

The refractive index of silica glass n_s_ core changes with wavelength λ in unite of µm according to the Sellmeier equation (1)^18–23^:

1 $$ n_{s} = \sqrt {1 + \frac{{a_{1} \lambda^{2} }}{{\lambda^{2} - b_{1}^{2} }} + \frac{{a_{2} \lambda^{2} }}{{\lambda^{2} - b_{2}^{2} }} + \frac{{a_{3} \lambda^{2} }}{{\lambda^{2} - b_{3}^{2} }}} $$

where a_1_, a_2_, a_3_, b_1_, b_2_, and b_3_ represent Sellmeier coefficients as mentioned in references^24–26^.

Drude-Lorentz model was employed for material dispersion of Au as shown below in Eq. (2)^27^:

2 $$ \varepsilon_{m} = \varepsilon_{\infty } - \frac{{\omega_{D}^{2} }}{{\omega (\omega + i\gamma_{D)} }} + \frac{{\Delta_{\varepsilon } \Omega_{L}^{2 } }}{{\left( {\omega^{2} - \Omega_{L}^{2} } \right) - i\Gamma_{L} \omega }} $$

The variables of Eq. (2) is listed in Table 1^27^.

**Table 1:** The variables of Drude-Lorentz model for gold layer of Eq. (2)^27^.

Variables	Description	Value	Unite
$\varepsilon_{\infty }$	Permittivity	5.9673	–
$\Delta_{\varepsilon }$	Weighting vector	1.09	–
$\omega$	The angular frequency	–	–
$\omega_{D}$	Plasma frequency	*ω*_*D*_/2π = 2113.6	THz
$\gamma_{D}$	Damping frequency	*γ*_*D*_/2π = 15.92	THz
$\Omega_{L}$	Frequency of the Lorentz oscillator	*ω*_*L*_/2π = 650.07	THz
$\Gamma_{L}$	The spectral width of the Lorentz oscillator	*γ*_*L*_/2π = 104.86	THz

**Figure 1:**
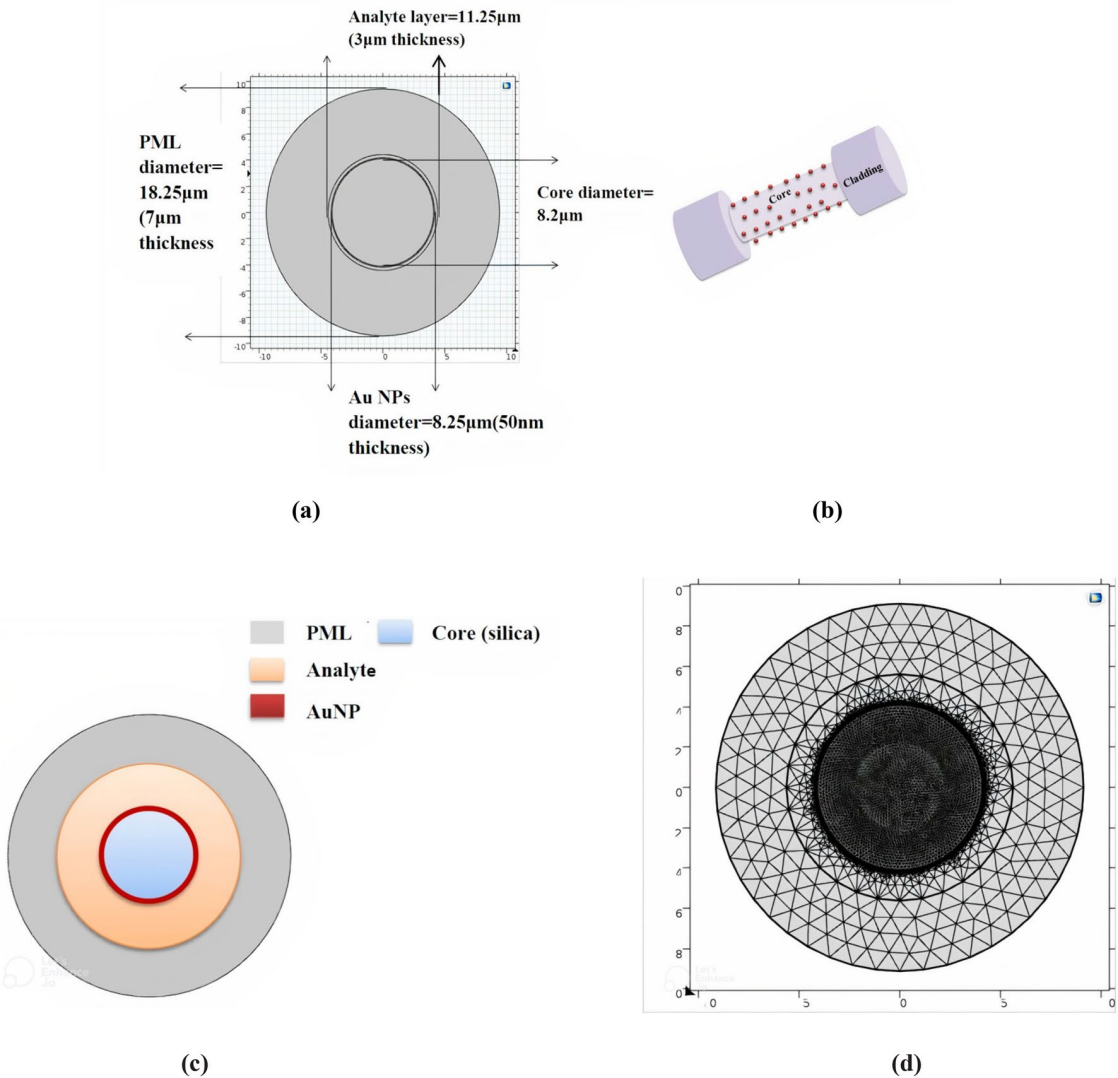
Presents (**a**) the cross section and the dimension of the sensor final structure, (**b**) the geometry of the unclad fiber deposited with Au NPs, (**c**) the core, Au NPs layer, analytic layer, and PML materials, and (**d**) the mesh of the sensor.

COMSOL Multiphysics 5.1 Finite element method (FEM) is used to design the sensor. After opening the program and choosing the space dimension to be two dimensional model (2D), the physics is chosen to be Electromagnetic wave, frequency domain (ewfd) and the study type is chosen to be mode analysis. Then, the geometries, the materials, and mesh type are also selecting to measure the confinement loss of the sensor from the imaginary part of the effective refractive index n_eff_ of the fundamental mode. Figure 1a and b shows cross section and the geometry of the sensor, respectively while Fig. 1c, show the materials of the core and perfectly matched layer (PML) which is made of silica, Au NPs, and the analytic layer which first filled with air (empty fiber), then filled with the liquids mentioned before. Figure 1d shows the mesh of the designed sensor.

Table 2 below presents all the dimensions and parameters of the proposed sensor.

**Table 2:** Presents the parameters of the sensor.

Name	Value	Description
d_co	8.2 µm	Core diameter
d_Au	8.25 µm	Gold diameter
d_analyte	11.25 µm	Analyte diameter
PML	18.25	Perfectly matched layer
Wl	300–1000 nm	Operating wavelength
Fo	c_const/wl[nm]	Operating frequency
a1	0.696163	Sellmier coefficient
a2	0.4079426	Sellmier coefficient
a3	0.897479400	Sellmier coefficient
b1	4.67914827 × 10^–3^ (μm)^2^	Sellmier coefficient
b2	1.35120621 (μm)^2^	Sellmier coefficient
b3	97.9240025 (μm)^2^	Sellmier coefficient
n_s	Equation (1)	Sellmier equation
$\varepsilon_{\infty }$	Table 1	Permittivity
$\Delta_{\varepsilon }$	Table 1	Weighting Vector
$\omega$	Table 1	The Angular Frequency
$\omega_{D}$	Table 1	Plasma Frequency
$\gamma_{D}$	Table 1	Damping Frequency
$\Omega_{L}$	Table 1	Frequency of The Lorentz Oscillator
$\Gamma_{L}$	Table 1	The Spectral Width of The Lorentz Oscillator
$\varepsilon_{\infty }$	Table 1	Permittivity
$\varepsilon_{m}$	Equation (2)	Drude-Lorentz model Equation
n_air	1.000281	Air refractive index
n_NaCl	1.35	NaCl refractive index
n_Sucrose	1.3710	Sucrose refractive index
n_Glycerol	1.39	Glycerol refractive index

## Experiment work

**Figure 2:**
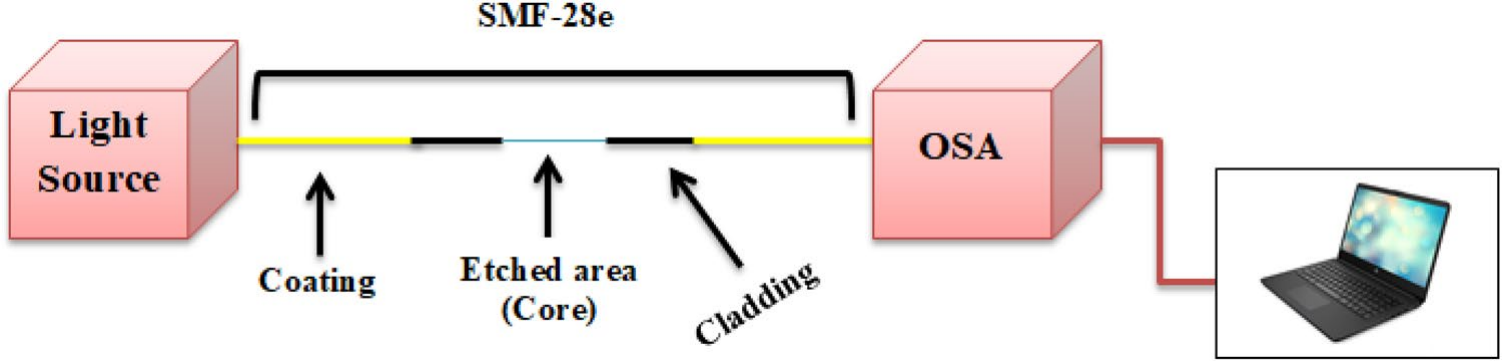
Shows the experimental setup.

Figure 2 presents the experimental setup of the proposed sensor.

### The preparation of gold nanoparticles (Au NPs)

**Figure 3:**
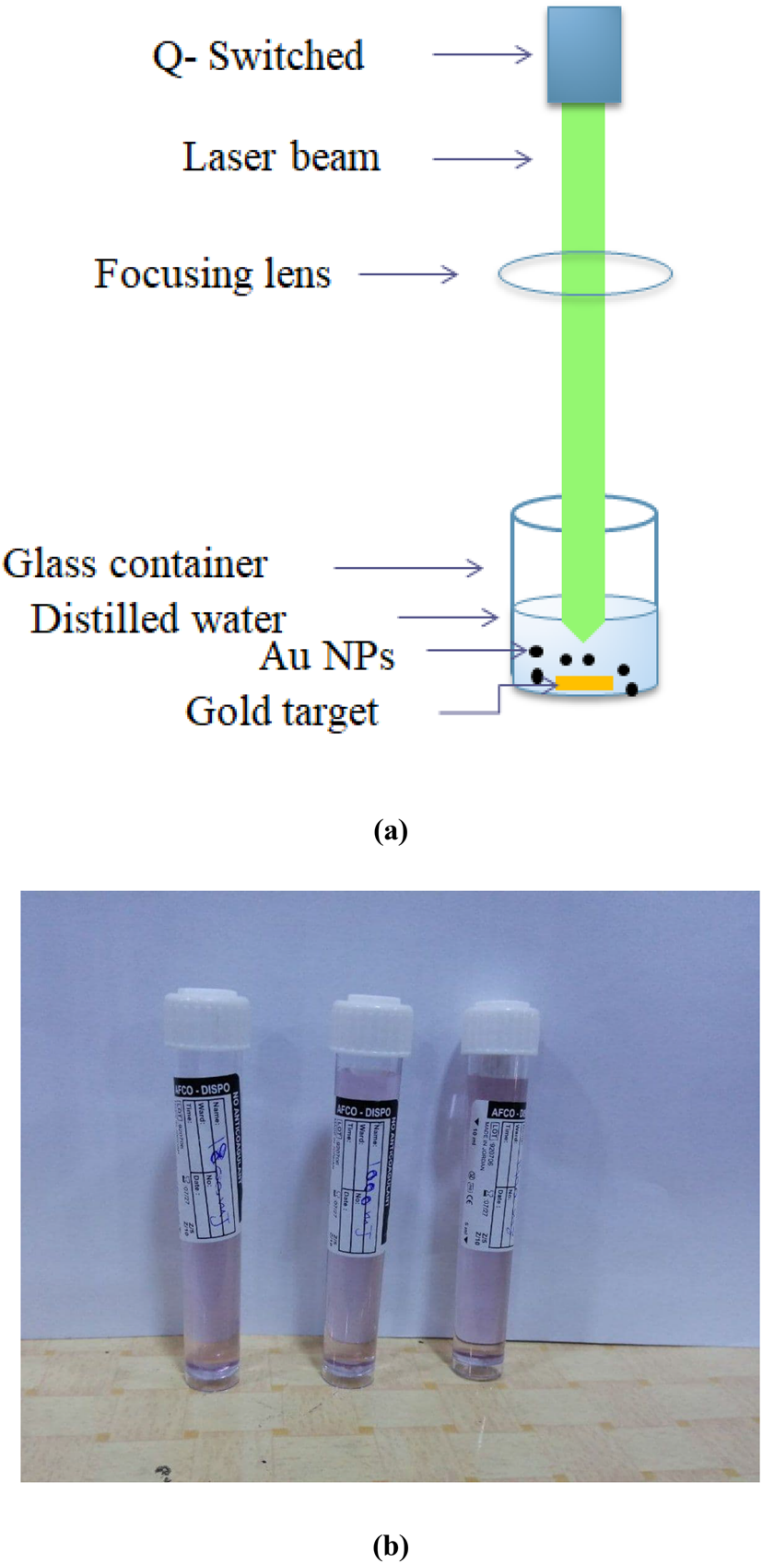
Presents: (**a**) a schematic of the PLA technique used to prepare the Au NPs, and (**b**) the prepared Au NPs at the three ablated energies.

PLA method was used to prepare the Au NPs. Gold sample of circular shape, high purity, and 3 cm diameter was immersed in plastic container filled with 10 ml of distilled water. In order to irradiate the gold sample, Laser Nd-YAG was placed at height of 12 cm from the sample as the focal length of the lens is 12 cm was used at wavelength of 532 nm and frequency of 0.3 (p/s). The gold sample was exposed to three ablated energy (1000, 1400, and 1800) mJ. The Au NPs was obtained and the distilled water was gain a violet color. Figure 3a) shows a schematic of the PLA technique for Au NPs while Fig. 3b) shows the obtained Au NPs.

X-ray diffraction (XRD) and transmission electron microscopy (TEM) were used to examine the surfaces of all samples. X-ray diffraction analysis was used to determine the phase composition of the nanoparticles using a diffractometer system (x′ pert pro MRD PW3040 Panalytical Company, Netherlands) equipped with Cu-K α-radiation of wavelength = 0.15418 nm at 40 kV and 30 mA. AFM was carried out by (SPM-9600, Scanning Probe Microscope, Shimadzu, Japan). A JEM 1200 EX II (Jeol Co., Japan) was used to describe the TEM.

### The preparation of the samples

Three solution samples were prepared which they are Sodium Chloride (NaCl), Glycerol and Sucrose solutions. Sodium chloride (NaCl) is an ionic compound composed of sodium and chloride. The resulting compound is a salt that is both commercially and biologically important. It has a crystalline structure, is solid, and has no odour. NaCl has a molar mass of 58.44 g per mole (g/mol)^28^.

Glycerol is a simple trihydric alcohol that appears as a clear, odourless, viscous liquid and nontoxic with a sweet taste. It is also naturally hygroscopic. C_3_H_8_O_3_ is the chemical formula, and the molar mass is 92.094 g/mol^29^.

Sucrose is a carbohydrate. Carbohydrates are primarily made up of carbon, hydrogen, and oxygen. Sugars are both water soluble and crystalline. Sugar involves glucose, fructose, sucrose, and lactose. Sucrose's chemical formula is C_12_H_22_O_11_, and its molar mass is 342.30 g/mol^30^.

A magnetic stirrer was used to mix the three samples with de-ionized (DI) water at 25 °C for 10 min. The resultant solutions are NaCl-DI water, Sucrose-DI water, and glycerol-DI water. Each of the solution has different refractive index. These three solutions have been chosen as they consider as an important biological analytes, availability, and cheap price.

Sodium Chloride (NaCl) solute from gain land chemical company (Purity = 99.9% molar mass = 58.44 g/mol), the Glycerol solute from fismer lecithin gmbh Company (purity = 99.9%, molar mass = 92.094 g mol^−1^) and Sucrose solute from Panreac Company (purity = 99.9%, molar mass = 342.29 gm/mol), are all diluted with concentration of 30% in water.

### Material and parameters

The main part of the experiment which was used to design the sensor is the standard single-mode fiber SMF-28e. This fiber has the following physical parameters: a core diameter of 8.2 m, a cladding diameter of (125 ± 0.7) µm, a mode field diameter of $\sim$ 10.4 µm, and a coating diameter of (245 ± 5) µm. Pure silica glass was used for the cladding as well as the core.

A laser beam with a wavelength of 415 nm and a power of 5 mw was used to deposit the gold NPs on the fibre core using a laser-induced method. The applied laser beam was expanded along the etched section using a beam expander. To track the RIs' sensing of prepared solutions, an optical spectrum analyzer (OSA) was used. The OSA has a wavelength range of 200 to 1000 nm.

### The deposition process

**Figure 4:**
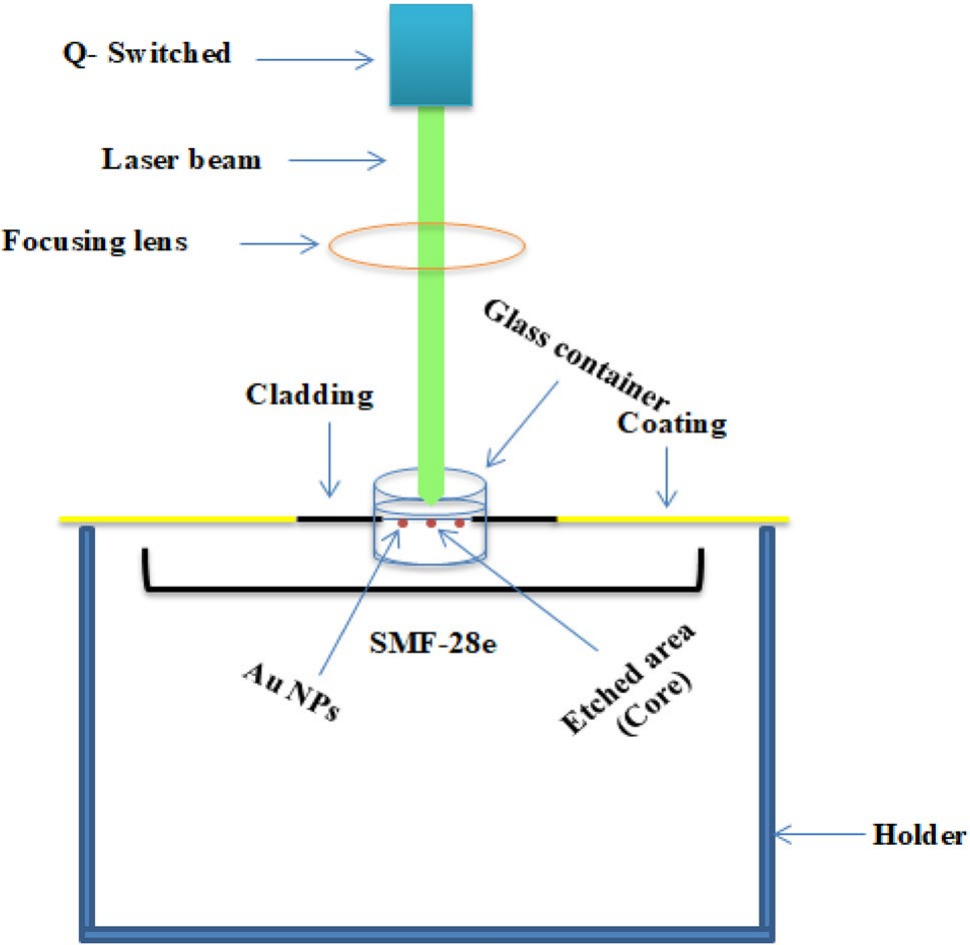
Presents a schematic of the deposition process of Au NPs.

A laser source with wavelength of 405 ± 10 nm was applied to the etched area of the SMF-28e of time duration of $\sim$ 4 h. This process is done during the immersed of the fiber in AuNPs to form a coated region on the etched region. Figure 4 shows the deposition process.

## Results and discussion

### Structural properties

**Figure 5:**
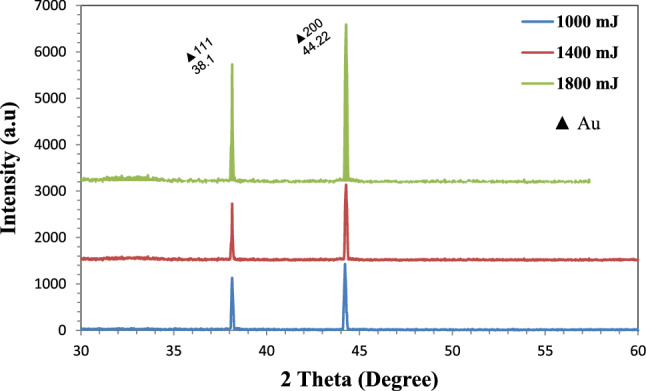
Presents the XRD analysis at wavelength of 532 nm for Au NPs.

The prepared Au NPs are analyzed using the XRD technique to support the desired wavelengths of working and structural properties after deposition method. The XDR pattern of Au NPs prepared at a 532 nm wavelength and using the three ablation energies (1000 mJ, 1400 mJ and 1800 mJ) is shown in Fig. 5. Two peaks with distinct intensities were seen at $2\theta =38.1^\circ$ and $2\theta =44.22^\circ$, respectively, and they belonged to the face-centered cubic (fcc) lattices (111) and (200). The best results were obtained at an ablated energy of 1800 mJ, indicating a high structure crystallization as a result of the uniform distribution of tiny Au NPs produced at this energy. Additionally, as laser ablation energy increased, the XRD pattern's peak intensities increased due to an increase in grain size and material concentration as well as an improvement in crystal quality at 1800 mj^31^.

### Gold nanoparticles morphology

**Figure 6:**
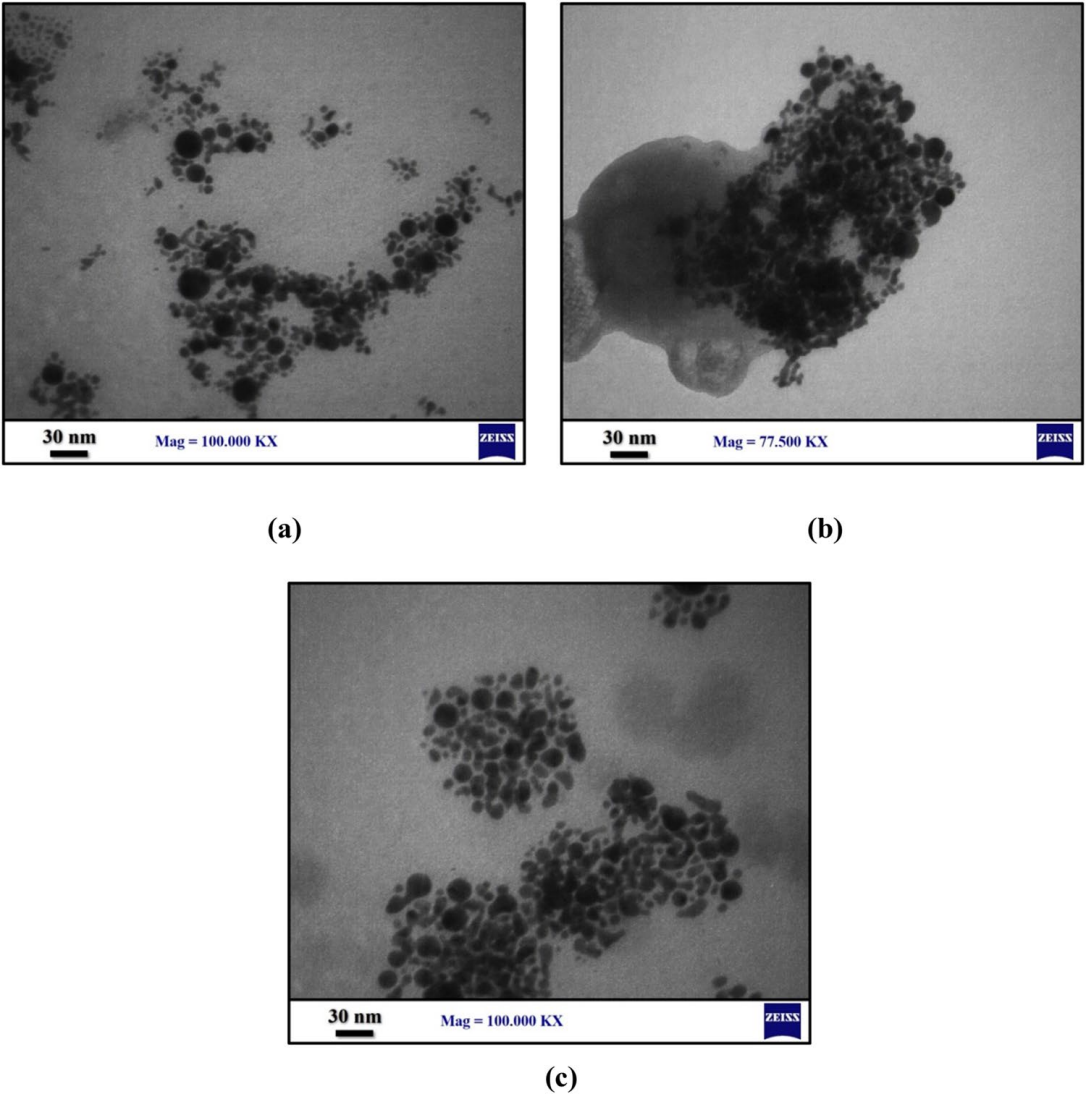
TEM of Au NPs at ablated wavelength of 532 nm and at ablated energy of (**a**) 1000 mJ, (**b**) 1400 mJ and (**c**) 1800 nm.

Transmission electron microscopy (TEM) and energy-dispersive X-ray spectroscopy (EDX) are required to investigate the morphological characteristics and size of Au NPs. The TEM images of Au NPs created using laser ablation with energies of 1000 mJ, 1400 mJ, and 1800 mJ are shown in Fig. 6a,b and c show the TEM images of Au NPs prepared at 532 nm wavelength of laser ablation of energies 1000 mJ, 1400 mJ and 1800 mJ, respectively. Figure 6a shows the accurate size distribution of Au NPs at ablated energy of 1000 mJ to be 5 nm and characterized by diameters in the range from 5 to 27 nm while the mean average diameter of 13 nm. Figure 6b shows the size distribution of Au NPs at ablated energy of 1400 nm to be 4.582 nm where the diameter ranging from 4 to 25 nm while the mean average diameter of 16 nm.Whilst, Fig. 6c shows the size distribution at ablated energy of 1800 mJ to be 3 nm with diameter ranging from 3 to 21 nm while the mean average diameter of 15 nm. It is obviously that as the laser ablation energy increased, the particles started to agglomerate. As a result, the ablation energy is vital in the deposition of Au NPs.

**Figure 7:**
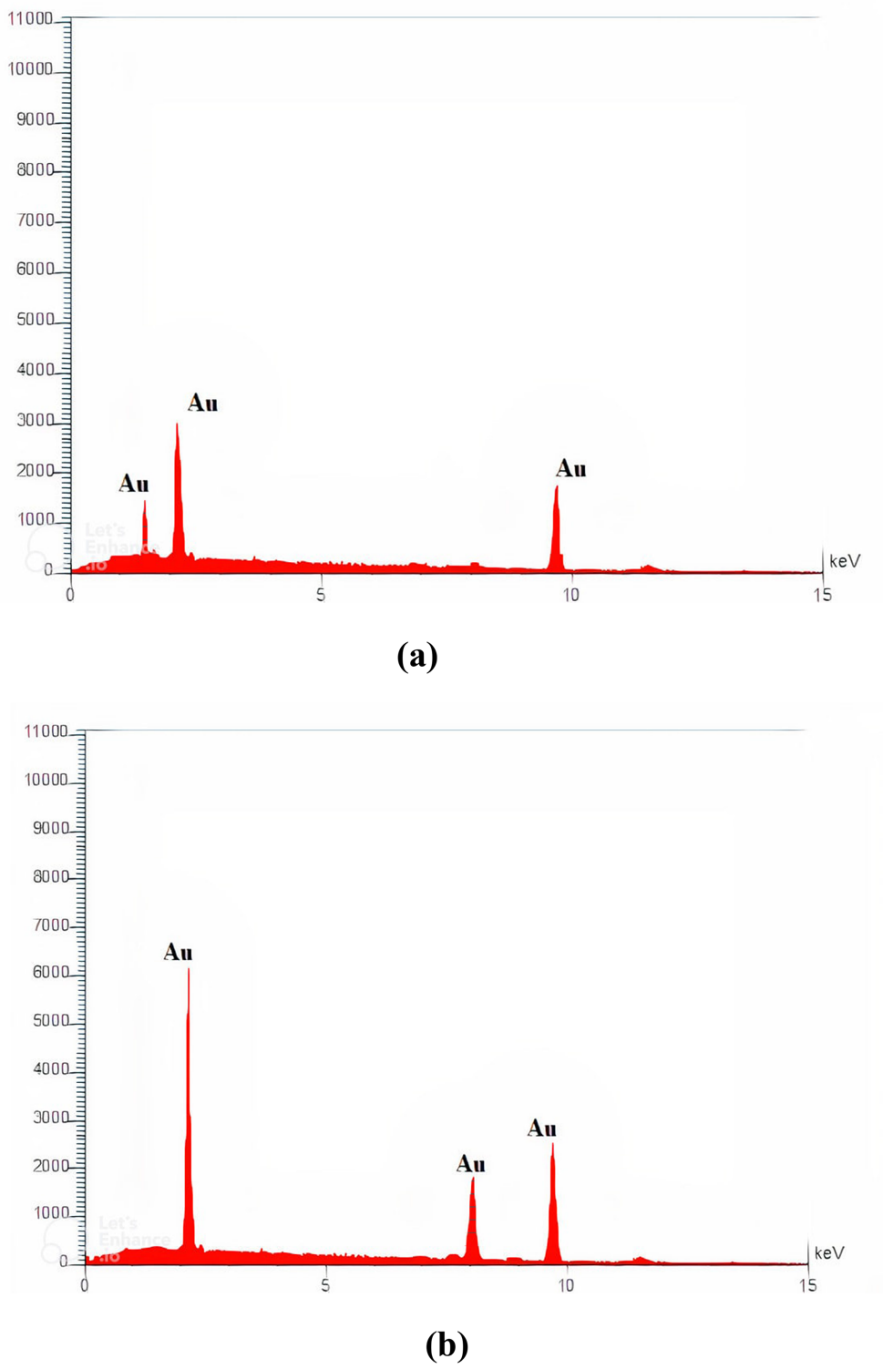
Presents Energy-dispersive X-ray spectroscopy (EDX) of Au NPs prepared at wavelength of 532 nm and ablated energy of (**a**) 1400 mJ, and (**b**) 1800 nm.

To determine the samples' chemical composition (Au NPs), EDX analysis was used^32^. With peaks at 1.5, 2.2, and 9.7 keV, the EDX spectrum in Fig. 7a at an ablated energy of 1400 mJ demonstrates the presence of Au NPs in the solution prepared at 532 nm. The strongest indication of the presence of Au is shown in the figure at 2.2 keV. Figure 7b shows an EDX spectrum at 1800 mJ of ablated energy, indicating the presence of Au at 2.2, 8, and 9.7 keV. At 2.2 keV, the signal is strongest.

### Optical properties

#### Photoluminescence (PL)

**Figure 8:**
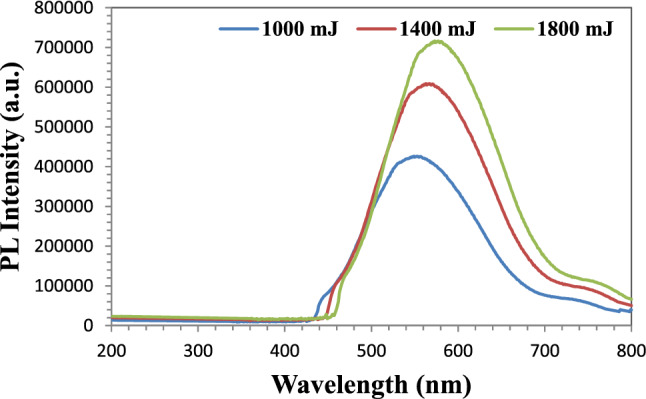
Photoluminescence (PL) properties of the prepared Au NPs.

The photoluminescence spectra for the prepared Au NPs at three laser ablation energies are shown in Fig. 8. Equation (2) was used to determine the incident photon's energy (E_g_) as a function of wavelength (λ)^31^.

3 $$ E_{g} = \frac{1240}{\lambda } $$

For each of the three ablated energies, three peaks were visible. Au NPs prepared with 1000 mJ of ablated energy produced the first peak. This peak's wavelength was 545 nm, and its energy gap was 2.27 eV. The second peak was for Au NPs produced at 1400 nm of ablated energy. The peak's wavelength and E_g_ value were 555 nm and 2.23 eV, respectively. The final peak was discovered at a wavelength of 567 nm and under an ablated energy of 1800 mJ. E_g_ = 2.18 eV is the energy gap for this peak. From the earlier findings, it is clear that the energy gap decreased and the wavelength shifted toward the red as the ablated energy increased. The large particle size is the cause of this. This large particle size will be eliminated as the ablated energy is increased, resulting in a red shift in wavelength.

#### Ultraviolet–visible

**Figure 9:**
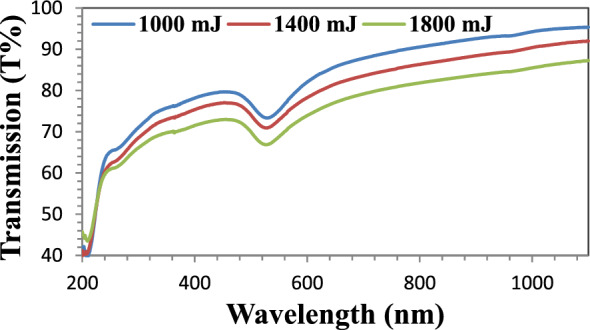
Transmission spectra of pure AuNPs at three laser ablation energies.

In order to examine the size and shape of the Au NPs solution over a wavelength range extending from 200 to 1100 nm and under three ablated energy conditions (1000 mJ, 1400 mJ and 1800 mJ), the UV–Vis Transmission measurement was used. The percentage of Au NPs transmission spectra is shown in Fig. 9. Under ablated energies of 1000 mJ, 1400 mJ, and 1800 mJ, the transmission percentages were 95.22%, 91.74%, and 86.96%, respectively. It can be demonstrated that for the three peaks, Au NPs started to respond more strongly at 518 nm. The maximum absorbance of the Au NPs solution was seen at this wavelength.

**Figure 10:**
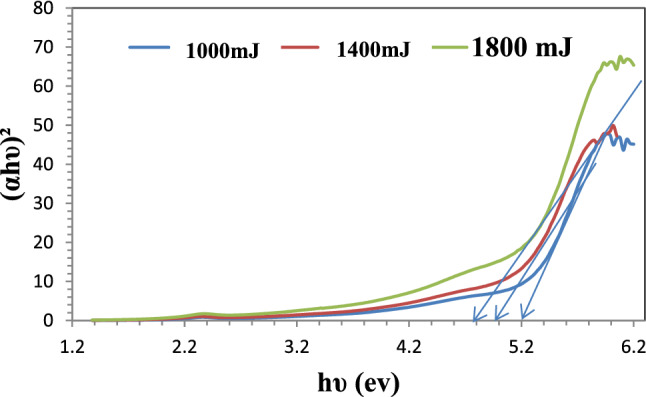
Shows photon energy versus $\left( {{\alpha h}\nu } \right)^{2}$.

Figure 10 shows the relationship between the photon energy and $\left( {{\alpha h}\nu } \right)^{2}$ of the AuNPs in the solution at three laser ablation energies (1000, 1400 and 1800) mJ to determine the values of the optical energy band gap E_g_. As can be seen, AuNPs received bandgap energy with a value equal to Eg = 5.2 eV at an ablated energy of 1000 mJ. Eg = 5 eV while at 1400 mJ. Eg = 4.8 eV at 1800 mJ.

### Output results and output spectra of the sensor obtained by OSA

#### Fiber-optic sensor without Au NPs

**Figure 11:**
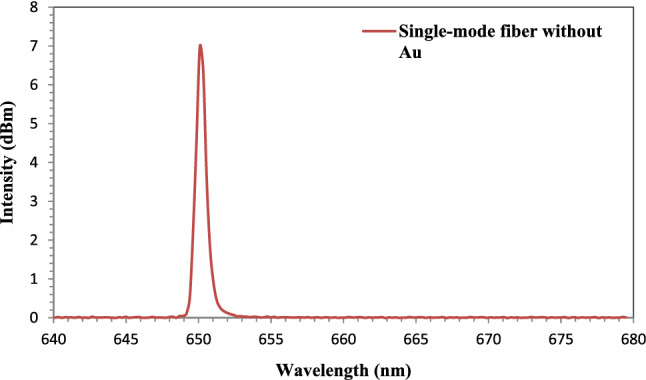
Output spectrum of the fiber-optic sensor without Au NPs.

The SMF-28e fiber-optic sensor configuration that is being suggested has the sensing component in the middle of the fiber. The sensing component had a length of 3 cm and was immersed in various solutions of sodium chloride, glycerol, and sucrose. Figure 11 demonstrates that the sensor had no effect on the signal propagating in the fiber core or on the wavelength (650 nm) before adding Au NPs. The TIR Propagation mechanism is therefore ineffective.

#### Fiber-optic sensor with Au NPs

**Figure 12:**
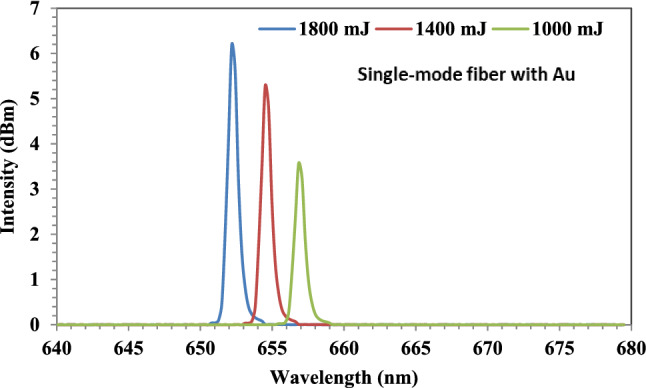
The output spectra of the fiber-optic with Au NPs at ablated energies of 1000, 1400, and 1800 mJ.

After using HF to remove the middle section of the cladding and replacing it with Au NPs before adding the analytes, a red shift in wavelength was observed in the output spectra, which are shown in Fig. 12. As can be seen, the wavelength was changed from 650 nm to almost 657 nm at 1000 mJ of ablated energy, and the intensity was 3.57dBm. On the other hand, the wavelength was changed to 654 nm and 652 nm at 1400 mJ and 1800 mJ, respectively, and the intensity was 5.28dBm and 6.19dBm. Because Au NPs are more sensitive than SiO_2_ and were deposited in the core, the intensity of the sensor increased. Additionally, it was evident that the wavelength shifted to the blue as the ablated energy increased from 1000 to 1800 mJ. This is due to both the high intensity at short wavelengths and the altered size of the Au NPs after altering the ablated energy.

**Figure 13:**
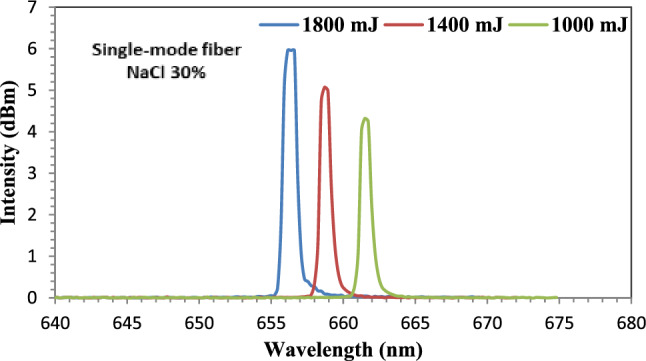
The output spectra of the fiber-optic immersed in NaCl solution at ablated energies of 1000, 1400, and 1800 mJ.

Figure 13 represents the output spectra of an unclad fiber-optic that was deposited with Au NPs and immersed in a 30% NaCl solution. It can be seen that red shifting from 650 to 661 nm at an ablated energy of 1000 mJ. On the other hand, a blue shift was taken place as the ablated energy was increased to 1400 mJ and 1800 mJ to 658 nm to 656, respectively. The highest intensity was obtained at 1800 mJ with value of 5.96dBm. The flat-top in the peak of intensity at 1800 mJ with nearly 1 nm indicates that the nanoparticles have uniform distribution and small size, resulting in better nanoparticle attachment to the sensing region.

**Figure 14:**
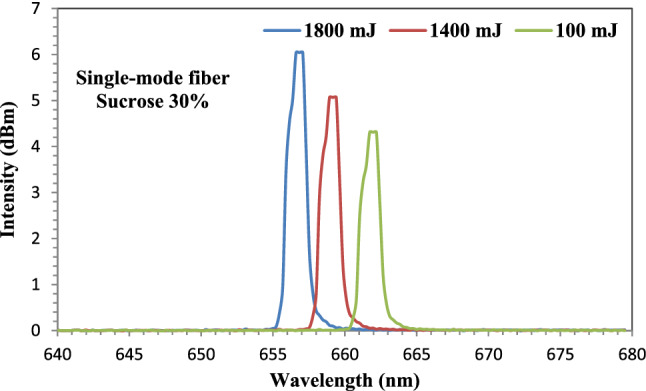
The output spectra of the fiber-optic immersed in Sucrose solution at ablated energies of 1000, 1400, and 1800 mJ.

Figure 14 represents the output spectra of an unclad fiber-optic that was immersed in a 30% sucrose solution. A blue shift in wavelength was observed as the laser ablation energies increased from 1000 to 1400 mJ and 1800 mJ. The shift was from 661 nm at 1000 mJ to 658 nm and to 654 nm at 1400 and 1800 mJ, respectively. The highest intensity was obtained at 1800 mJ with value of 6.04dBm. For the three spectra of the three ablated energies, a flat-top with 1 nm appeared. This is due to the uniform distribution of the Au NPs, which results in a high attachment to the sensing medium and an increase in sensitivity.

**Figure 15:**
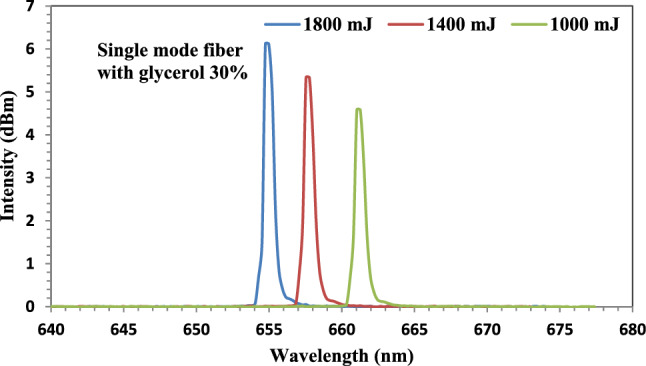
The output spectra of the fiber-optic immersed in Glycerin solution at ablated energies of 1000, 1400, and 1800 mJ.

Figure 15 represents the output spectra of an unclad fiber-optic immersed in a 30% concentration Glycerol solution. The graph depicts a red shift at ablated energies of 1000 mJ from650 to 661 nm. But, as the ablated energy increased to 1400 and 1800 mJ, a blue shift was taken place where the shift was to 657 nm and to 654 nm, respectively. The highest intensity was obtained at 1800 mJ with value of 6.11dBm.

### Simulation results of USMFOS with Au NPs layer

Light propagation in a fiber can produce evanescent waves, which can be transmitted to the fiber's external surface. This could have happened because of the fiber optic structure where it is the unclad fiber optic in this work. The key parameters for this study are the thickness of Au NPs, the wavelength, and the analyte RIs. The thickness of the Au NPs was changed from 40 to 100 nm in step of 10 nm. Then, since the best performance was gotten at 50 nm thickness, it was chosen to design the sensor. The sensor was designed with a 50 nm thickness because it had the best performance. At this thickness, the sensitivity was higher and the scattering of the mode were less. Furthermore, this thickness of Au NPs layer can be used in real experimental work. Then, the wavelength and the RIs which were change for each liquid and n_eff_ was obtained. Then, the confinement loss (${\alpha }_{CL}$) in (dB/m) was measured since it depends on the imaginary part of n_eff_ Im(n_eff_) of the fundamental core mode and the wavelength (λ) in µm as in Eq. (4)^33–35^

4 $$ \alpha_{CL} = 8.686 \times \frac{2\pi }{\lambda } \times {\text{Im}} \left( {n_{eff} } \right) \times 10^{6} \quad \left( \frac{dB}{m} \right) $$

**Figure 16:**
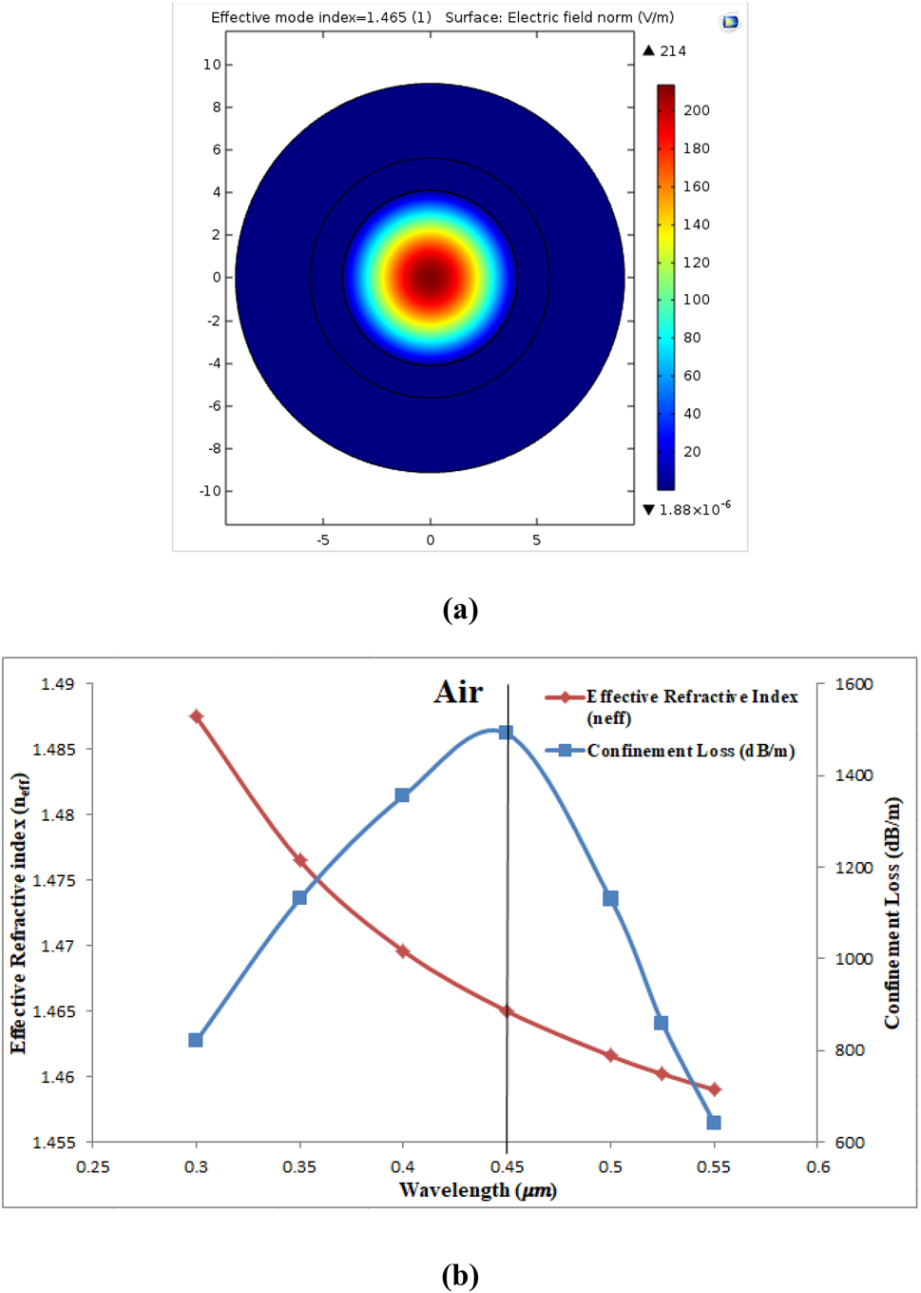
Presents (**a**) the fundamental mode of with air at 0.45 µm and (**b**) the confinement loss (dB/m) and n_eff_ curves of Air as a function of wavelength.

The unclad SMF was designed by laser transmission at wavelength range of (0.3–0.55) μm from the core of the fiber. The fiber was theoretically designed by using simulation program COMSOL Multiphysics 5.1 with core of silica glass RI derived from Eq. (1) and gold layer of thickness equal to 50 nm. The thickness of 50 nm was chosen after varying it from 30 to 60 nm in step of 10 nm and its effect on SPR and sensitivity of the sensor was observed. Figure 16a shows the electric field dispersion spectrum of the fundamental mode of air at λ = 0.45 μm at which the confinement loss reached its maximum and the electric field is confined in core of the fiber with n_eff_ = 1.465–1.23 × 10^−5^i. Figure 16b shows the ${\alpha }_{CL}$ (dB/m) which was calculated from Eq. (4) and Re(n_eff_) of the fundamental mode curves. It can be seen that the loss reached its maximum at λ = 0.45 µm and n_a_ of air equal to 1.000281 with value of 1491.37 (dB/m). Also in the same figure, it can be seen that n_eff_ was decreased as the wavelength increased. The SPR condition did not take place for air at this range of wavelength.

**Figure 17:**
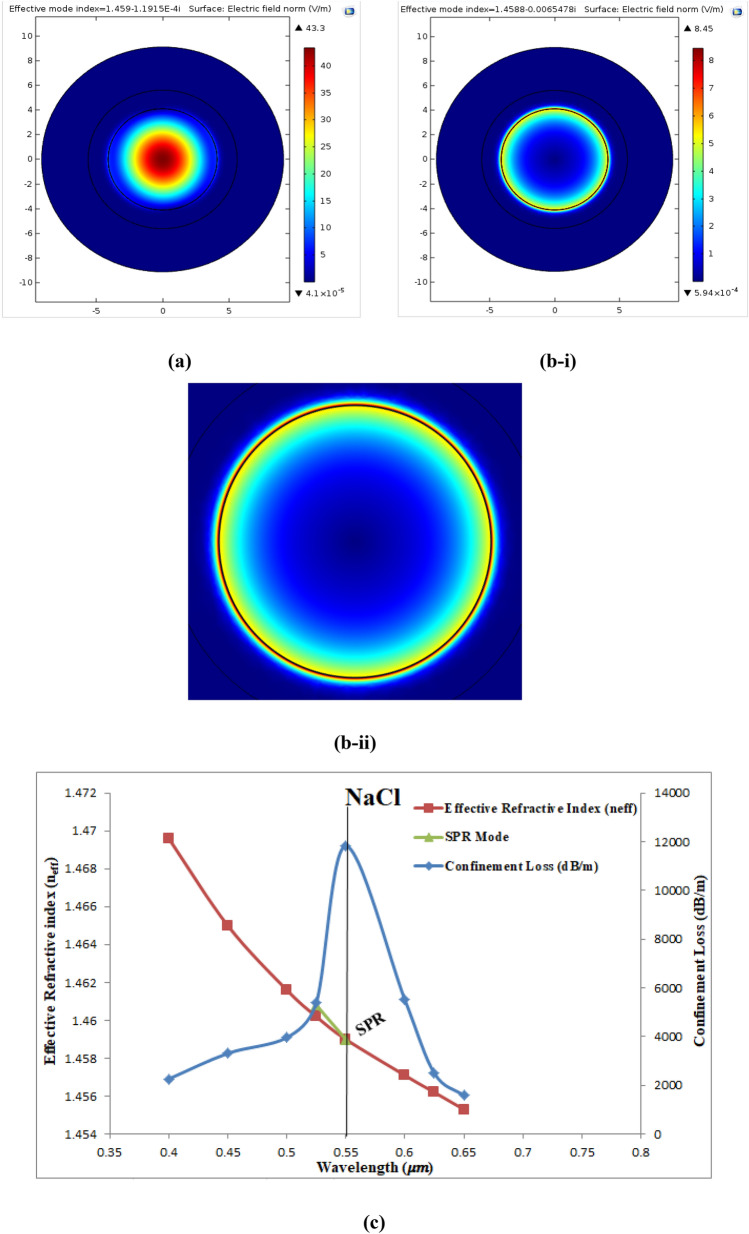
Presents (**a**) the fundamental mode of sucrose at 0.55 µm, (**b-i**) the SPR mode of NaCl-DI water at SPR wavelength of 0.55 µm, (**b-ii**) a magnified image for theSPR mode, and (**c**) Curves of the confinement loss (dB/m), Re(n_eff_) of the fundamental mode and Re(n_eff_) of SPR mode as a function of wavelength.

NaCl-DI water solution was used in our study to fill the analytic layer of the fiber. Figure 17a shows the electric field dispersion spectrum of the fundamental mode of this solution at wavelength of 0.55 µm with n_eff_ = 1.459–1.19 × 10^−4^i. Figure 17bi shows the SPR mode at SPR wavelength equal to 0.55 µm where Re(n_eff_) of SPR mode equal to1.4588 which is nearly equal to that of the fundamental mode. Figure 17bii shows a magnified image of the resonance mode. Figure 17c shows the ${\alpha }_{CL}$ (dB/m), Re(n_eff_) of the fundamental mode, and Re(n_eff_) of the SPR mode at wavelength range from (0.4–0.65) µm. It can be seen that the ${\alpha }_{CL}$ shifted towards longer wavelength of 0.55 µm and n_a_ of air equal to 1.35 with maximum value of 11,823.119 (dB/m) where the SPR condition was satisfied at this wavelength.

**Figure 18:**
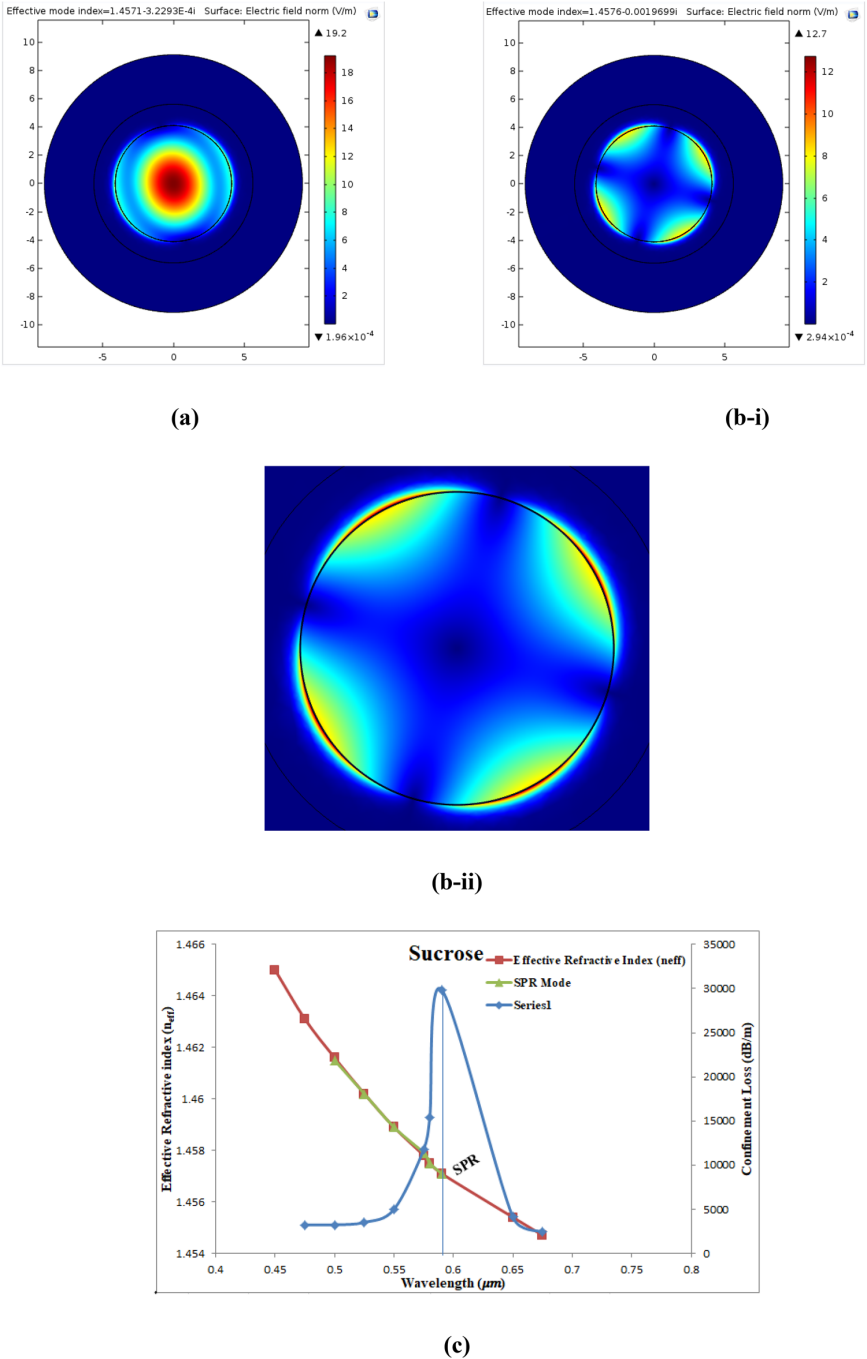
Presents (**a**) the fundamental mode of sucrose at 0.59 µm, (**b-i**) the SPR mode of sucrose at SPR wavelength of 0.59 µm, (**b-ii**) a magnified image for theSPR mode, and (**c**) Curves of the confinement loss (dB/m), Re(n_eff_) of the fundamental mode and Re(n_eff_) of SPR mode as a function of wavelength.

The sucrose composes of glucose, fructose, sucrose, and lactose. The increment in these substances above the reference level can cause sugar disease in human blood. Thus, the sucrose-DI water solution was used to fill the analytic layer of the fiber to test the proposed sensor theoretically. Figure 18a shows the electric field dispersion spectrum of the fundamental mode of sucrose-DI water sensor at λ = 0.59 μm where the electric field was completely confined in the core and had n_eff_ = 1.4571–3.32 × ^10−4^i.Fig. 18bi show the SPR mode at which the fundamental mode was coupled with the SPR mode and electric field distributed around Au NPs layer. The Re(n_eff_) of SPR mode equal to1.4576 which is nearly equal to that of the fundamental mode. Figure 18bii shows a magnified image of this mode. Figure 18c shows curves present the ${\alpha }_{CL}$ (dB/m), Re(n_eff_) of the fundamental mode, and Re(n_eff_) of the SPR mode, at wavelength range expands from (0.475–0.675) µm. It can be seen that the maximum value (29,871.50 dB/m) of this loss is shifted towards longer wavelength at 0.59 µm and n_a_ = 1.3710. The wavelength at which the ${\alpha }_{CL}$ reached its maximum is called the SPR wavelength because the SPR condition is satisfied at this wavelength.

**Figure 19:**
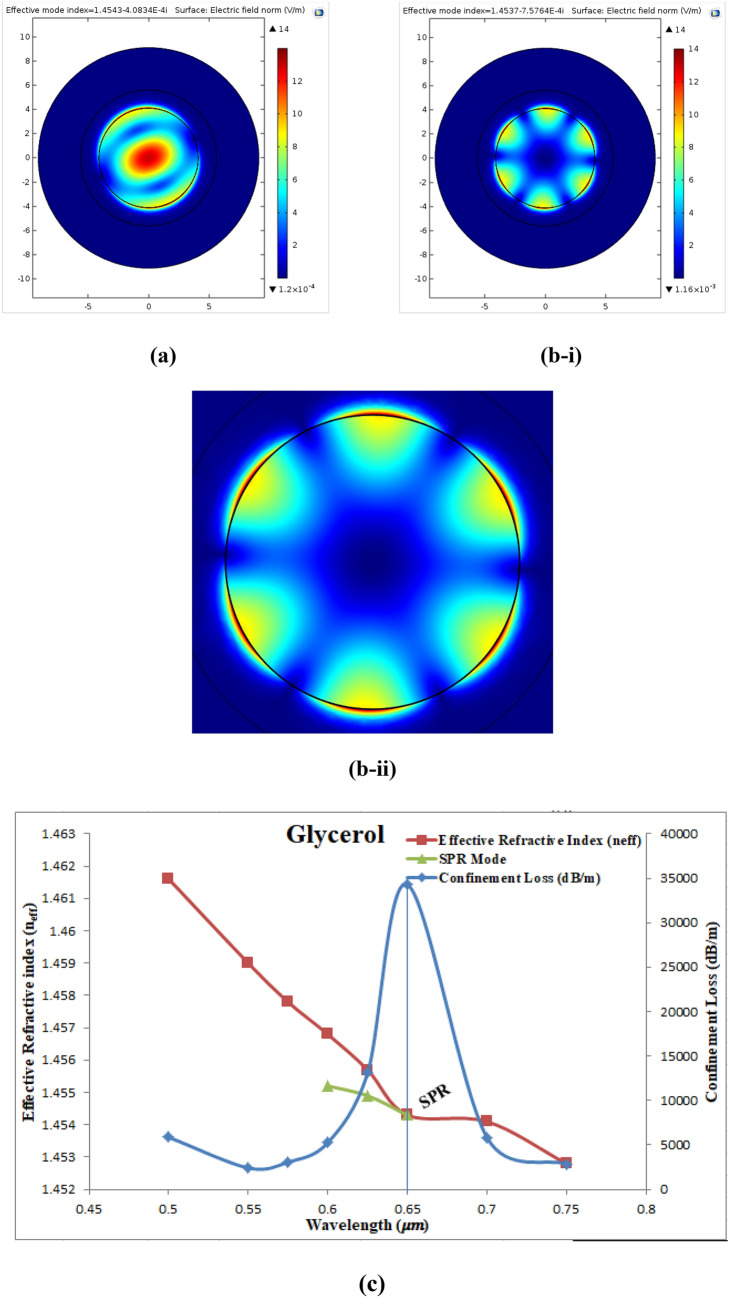
Presents (**a**) the fundamental mode of glycerol-DI water at 0.65 µm, (**b-i**) the SPR mode of glycerol-DI water at SPR wavelength of 0.65 µm, (**b-ii**) a magnified image for the SPR mode, and (**c**) Curves of the confinement loss (dB/m), Re(n_eff_) of the fundamental mode and Re(n_eff_) of SPR mode as a function of wavelength.

Glycerol-DI Water Solution to fill the analytic layer of the fiber. Figure 19a shows the electric field dispersion spectrum of the fundamental mode of glycerol-DI water at wavelength of 0.65 µm with n_eff_ = 1.4543–4.08 × 10^−4^i. Figure 19bi shows the SPR mode at SPR wavelength of 0.65 µm where Re(n_eff_) of SPR mode equal to1.4537 which is nearly equal to that of the fundamental mode. Figure 19bii shows a magnified image of the SPR mode. Figure 19c show the ${\alpha }_{CL}$ (dB/m), Re(n_eff_) of the fundamental mode, and Re(n_eff_) of the SPR mode at wavelength range from (0.4–0.75) µm. It can be seen that the ${\alpha }_{CL}$ shifted towards longer wavelength of 0.65 µm and n_a_ = 1.39 with maximum value of 34,285.40 (dB/m) where the SPR condition was satisfied at 0.7 µm.

**Figure 20:**
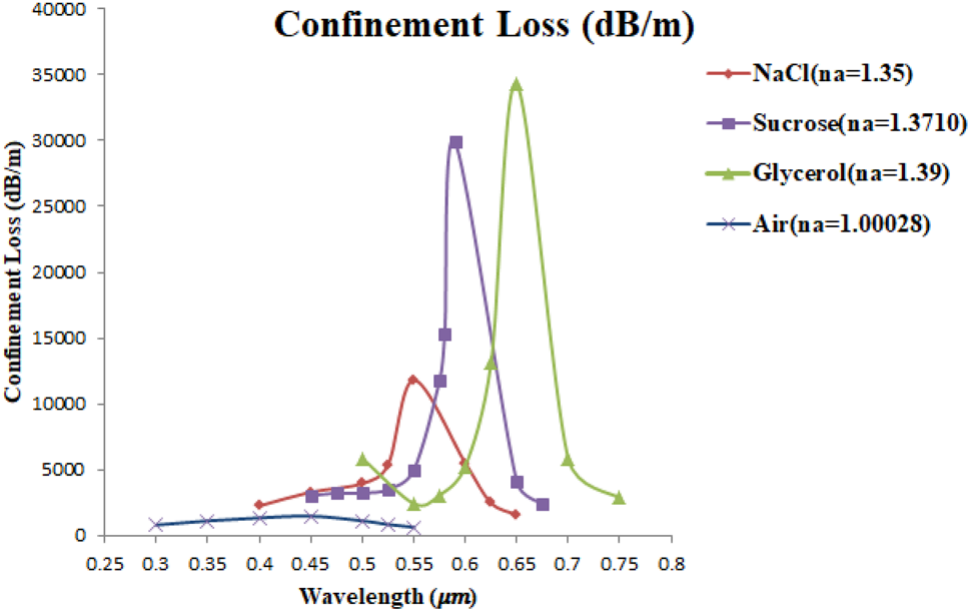
The confinement loss ${\alpha }_{CL}$ (dB/m) for air, NaCl-DI water, sucrose-DI water, and glycerol-DI water as a function of wavelength.

Figure 20 shows a relationship between the ${\alpha }_{CL}$ and a range of wavelength extended from 0.3 to 0.75 μm, for air, NaCl-DI water, sucrose-DI water, and glycerol-DI water. It can be seen that as n_a_ of these liquids were increased, ${\alpha }_{CL}$ was also increased and shifted towards longer wavelength. The cause of this phenomenon is that as the distance between the Au NPs and the core increases, the electric field at the longer wavelength can pass and couple with SPR modes on the Au NPs^27^.

**Figure 21:**
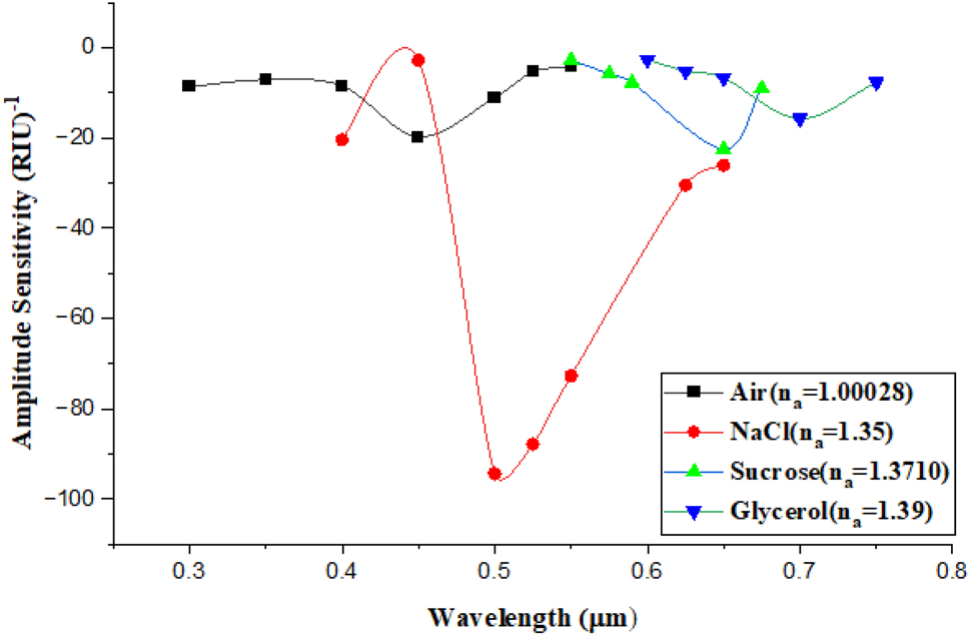
Presents the amplitude sensitivity of air, NaCl-DI water, sucrose-DI water, and glycerol-DI water as a function of wavelength.

In order to measure the performance of the proposed sensor, the amplitude sensitivity ${S}_{\lambda }$, The amplitude sensitivity ${S}_{a}$ in unite of [RIU]^−1^ is an important parameter in estimating the performance of the sensor and can be defined by Eq. (5)^27^:

5 $$ S_{a} = - \left( {\frac{{\Delta \alpha \left( {\lambda ,n_{a} } \right)}}{{\Delta n_{a} }}} \right)/\alpha \left( {\lambda ,n_{a} } \right)_{Cinitiated} \left[ {RIU} \right]^{ - 1} $$

where ${\alpha \left(\lambda ,{n}_{a}\right)}_{Cinitiated}$ represents the initial confinement loss, the analyte RI changes $(\Delta {n}_{a})$ and $\Delta \alpha (\lambda ,{n}_{a})$ is the variation between two losses. Figure 21 shows the resultant amplitude sensitivity of air, NaCl sucrose, and glycerol, at n_a_ = 1.000281, 1.35, 1.3710, and 1.39, respectively. The obtained ${S}_{a}$ were − 19.809, − 94.433, − 22.517, − 15.775 [RIU]^−1^, respectively.

The wavelength sensitivity ${S}_{\lambda }$ of the sensor is also important to measure the performance of the sensor. The sensitivity is calculated by recording the shift in the resonance wavelength peak $(\Delta {\lambda }_{peak}$) as the $(\Delta {n}_{a})$ changes and can be defined by Eq. (6)^27^:

6 $$ S_{\lambda } = \frac{{\Delta \lambda_{peak} \left( {n_{a} } \right)}}{{\Delta n_{a} }}\quad \left[ {\frac{nm}{{RIU}}} \right] $$

$\Delta {n}_{a}$ are (1.35–1.00028), (1.3710–1.35), and (1.39–1.3710) which are equal to 0.349719, 0.021, and 0.019 at $\Delta {\lambda }_{peak}$ equal to 100, 40, 50, and 60 for NaCl, sucrose, and glycerol, respectively. The wavelength sensitivity ${S}_{\lambda }$ was equal to 285.9435, 1904.762, and 3157.984737 (nm/RIU). It can be seen Eq. (6) that as $\Delta {n}_{a}$ decreases, the sensitivity ${S}_{\lambda }$ increases. Furthermore, we can see from the same equation that $\Delta {\lambda }_{peak}$ is directly proportional to ${S}_{\lambda }$. As a result, the maximum obtained ${S}_{\lambda }$ was for glycerol-DI water and it is not very large since $\Delta {\lambda }_{peak}$ is not very large.

Furthermore, the resolution $R$ in unite of [RIU] of the analyte RI is a crucial consideration for the sensor, and it is expressed in Eq. (7)^27^:

7 $$ R = \frac{{\left( {\Delta \lambda_{min} } \right)\left( {\Delta n_{a} } \right)}}{{\left( {\Delta \lambda_{peak} } \right)}} \quad \left[ {RIU} \right] $$

where 0.1 nm is assumed to be $\Delta {\lambda }_{min}$ of the minimum wavelength resolution. The calculated $R$ was 3.50 × 10^–4^, 5.25 × 10^–5^, and 3.16 × 10^–5^ [RIU] for NaCl-DI water, sucrose-DI water, glycerol-DI water, respectively.

Also, the sensing length ${L}_{s}(\lambda ,{n}_{a})$ in unite of [m] is another important performance for the sensor and is represents the inverse of the peak confinement loss at the SPR wavelength and can be defined in Eq. (8)^27^:

8 $$ L_{s} \left( {\lambda ,n_{a} } \right) = \frac{1}{{\alpha_{CL} }} \quad \left[ m \right] $$

**Figure 22:**
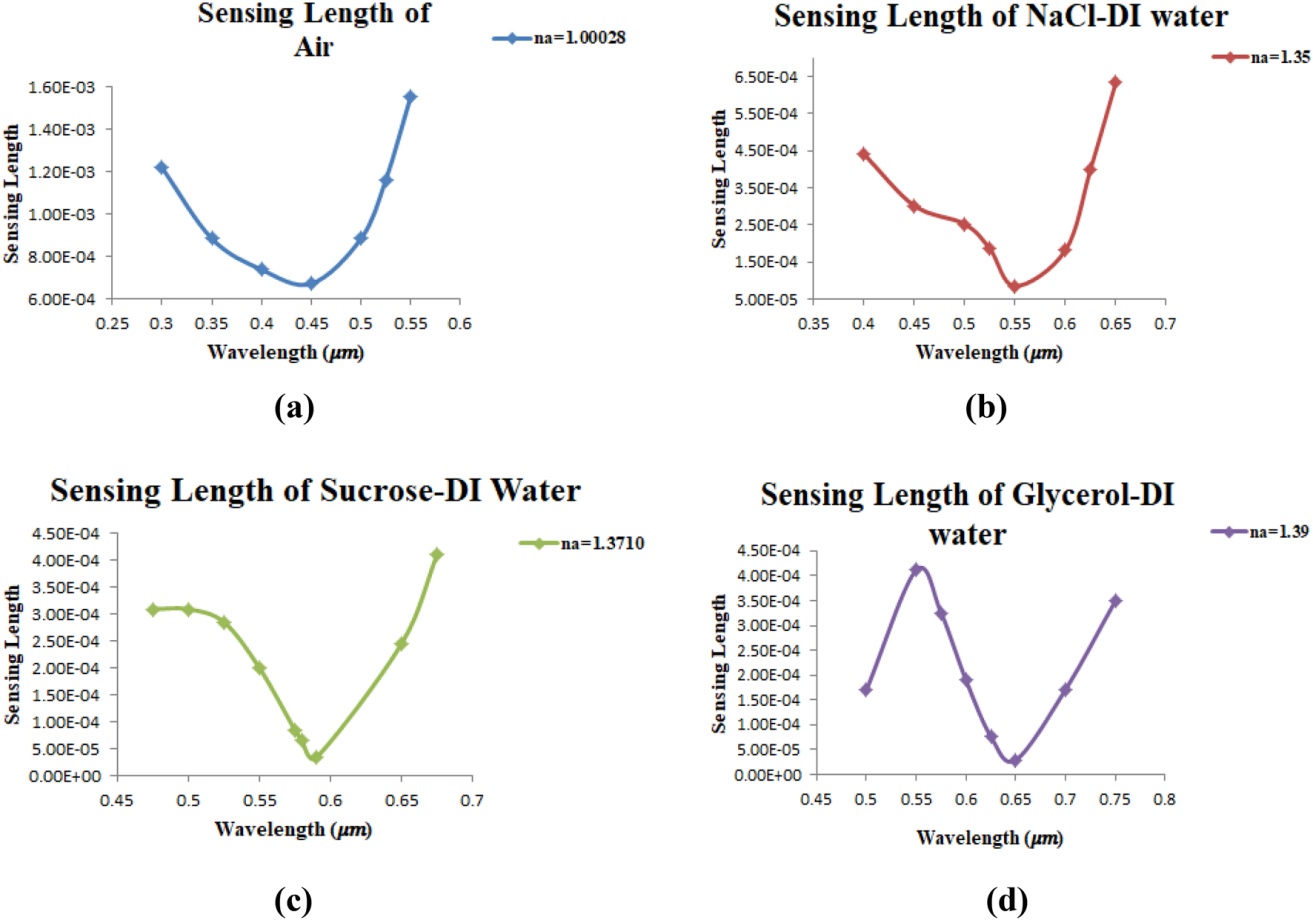
Presents the sensing length ${L}_{s}\left(\lambda ,{n}_{a}\right)$ of air, NaCl-DI water, sucrose-DI water, glycerol-DI water as a function of wavelength.

${L}_{s}\left(\lambda ,{n}_{a}\right)$ Was found to be 6.71 × 10^–4^, 8.46 × 10^–5^, 3.35 × 10^–5^, 2.29 × 10^–5^ (m) for air, NaCl-DI water, sucrose-DI water, and glycerol-DI water, respectively. It can be seen that as n_a_ increases, ${L}_{s}\left(\lambda ,{n}_{a}\right)$ decreases because of the increment in ${\alpha }_{CL}$ which is inversely proportional to ${L}_{s}\left(\lambda ,{n}_{a}\right)$. Higher losses cause higher fabrication complexity. Figure 22a–d, show curves of ${L}_{s}\left(\lambda ,{n}_{a}\right)$ for air, NaCl-DI water, sucrose-DI water, and glycerol-DI water, respectively. The highest confinement loss was obtained for glycerol-DI water with value of 34,285.40 (dB/m) where the sensing length for this solution was 2.29 × 10^–5^ (m).

**Figure 23:**
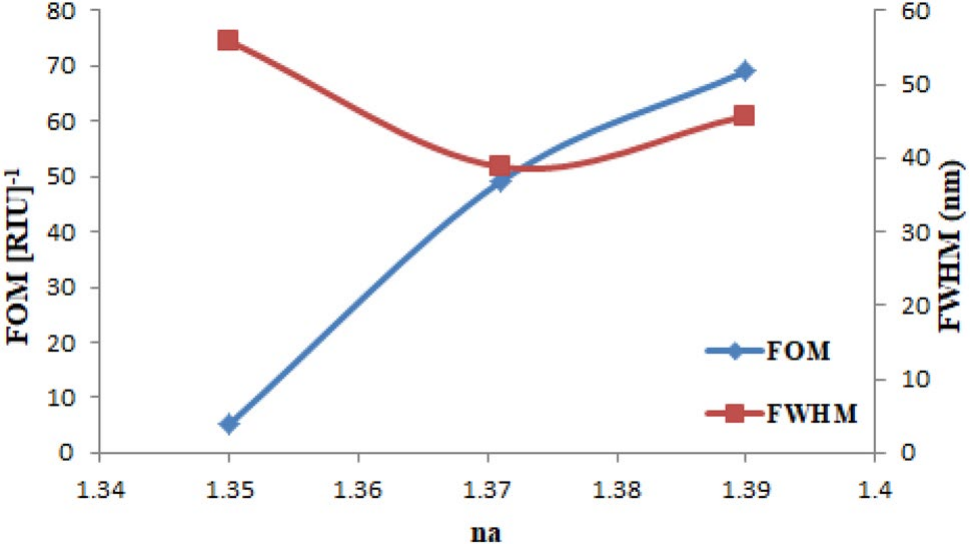
Presents the FOM and FWHM at n_a_ = 1.35, 1.3710, and 1.39 for NaCl-DI water, sucrose-DI water, and glycerol-DI water.

Furthermore, the Figure of Merit (FOM) was also measured in unite of [RIU]^−1^ which represents the overall performance of the sensor and is given in Eq. (9)^27^:

9 $$ FOM = \frac{{S_{\lambda } }}{FWHM} \left[ {RIU} \right]^{ - 1 } $$

where ${S}_{\lambda }$ is the wavelength sensitivity as defined in Eq. (6), and FWHM is the full width at half maximum of the resonance peak where the SPR curve broadens at half maximum. The calculated FOM [RIU]^−1^ for NaCl-DI water, sucrose-DI water, and glycerol-DI water is shown in Fig. 23. It was found to be 5.115, 49.066, and 69.055 [RIU]^−1^ at FWHM of 55.9, 38.82, and 45.73 nm, respectively. The FWHM should be low and the sensitivity should be high in order to produce high FOM. The maximum FOM value of 69.055[RIU]^−1^ is achieved for glycerol-DI water solution for analyte RI of 1.39 and FWHM of 45.73 nm as indicates in the figure below.

Table 3 presents a comparison between the presents work and previous works. The table shows different geometries of previously designed sensor which used either metal NPs or film to coat the fiber with different thicknesses. It can be seen that as the geometry of the fiber and the thickness of the deposited metal NPs affected the performances of the sensor.

**Table 3:** Presents a comparison between this work and previous work.

Geometry of the fiber	Nanomaterial/film	Thickness/diameter	Refractive index range	Sensitivity	Reference
Cladding removed U-bent plastic optical fiber	Spherical Au NPs	Radius = 10 nm	Not reported	Not reported	^36^
U-shaped fiber-optic	Au NPs	Diameter = 18 nm	1.333–1.387	~ 25.1[ΔA/RI]	^37^
Hetero-core structured fiber optic	Silver (Ag) thin film	40 nm thickness	Not reported	Not reported	^38^
Hetero-core structured fiber	Au NPs	Not reported	1.333–1.365	765 [nm/RIU]	^39^
Optical fiber sensor, based on a slab waveguide of bacterial cellulose (BC)	Au film	60 nm	1.332–1.350	Not reported	^40^
Cladding removed U-bent plastic optical fiber	Triangular and spherical Ag NP	Not reported	1.3317–1.3610	1116.8 [nm/RIU] and 342.7 [nm/RIU]	^41^
Side-polished D-type fiber	Au film	50 nm	1.334–1.388	3328.1 [nm/RIU]	^42^
This work	Au NPs	50 nm	1.000281–1.39	3157.98 [nm/RIU]	–

## Conclusion

In our work, the proposed unclad fiber-optic sensor is based on LSPR phenomenon and designed theoretically by using COMSOL Multyphysics 5.1 (FEM). Au NPs of 50 nm thickness was deposited on the core of the fiber in order to enhance the performances of the sensor. The fabrication of sensor is very simple and highly efficient on the SPR phenomenon. The outcomes of the sensor show that ${\alpha }_{CL}$ is shifted towards the longer wavelength as ${n}_{a}$ is increased. Furthermore, the Phase matching condition is satisfied at maximum loss. The phase matching condition is satisfied as Re(n_eff_) of the fundamental mode becomes nearly equal to that of the SPR mode and hence the SPR takes place. It was found that As the ${\Delta \lambda }_{peak}$ increased and $\Delta {n}_{a}$ decreased, the sensitivity increased as a result the maximum wavelength sensitivity was obtained for glycerol-DI water solution with value of 3157.98 [nm/RIU] and resolution of 3.16 × 10^–5^ [RIU] in RI range from 1.000281 to 1.39. The overall performances of the sensor are represented by the FOM which should be high to provide good performances for the sensor at low FWHM. It is shown that the sensor can be considered as biosensor to detect the glycerol-DI water solution. The sensor was also experimentally tested, XRD technique was used to study the structural properties of the prepared Au NPs and has shown that as the ablated energy increased, the intensity as well as the crystallization increased which provided at ablated energy of 1800 mJ. TEM shown that the average diameter of Au NPs wer13, 16, and 15 nm for the three ablated energies, respectively, while EDX spectrum indicates the presence of Au NPs in the prepared solution. PL and UV–Vis transmission both show that the band gap energy decreased as the ablated energy increased. The output results of the sensor show that before the deposition of Au NPs, there were no shift in wavelength and no effect on the propagated signal in the core of the fiber. Whilst, it has shown that there was a blue shift as the ablated energy increased from 1000 to 1800 mJ as well as the intensity has increased, after the deposition if Au NPs. Then, when the sensor was immersed in NaCl-DI water solution, sucrose-DI water solution, and glycerol-DI water solution, it was shown that best results were obtained for sucrose-DI water and glycerol-DI water solutions since that for the three spectra of the three ablated energies, a flat-top with 1 nm was appeared. This is due to the uniform distribution of the Au NPs, which results in a high attachment to the sensing medium and an increase in sensitivity. For theoretical and experimental results, it can be seen that this sensor provide performances that could be used for farther investigations in biological or chemical fields.

## Data Availability

Correspondence and requests for materials should be addressed to M.A. Fakhri, Sara M. Tariq, and E. T. Salim.
